# Population Viability Analysis of the Endangered Roan Antelope in Ruma National Park, Kenya, and Implications for Management

**DOI:** 10.1155/2018/6015694

**Published:** 2018-02-15

**Authors:** Johnstone K. Kimanzi

**Affiliations:** Department of Wildlife Management, University of Eldoret, P.O. Box 1125-30100, Eldoret, Kenya

## Abstract

Population viability analysis (PVA) was used to (1) establish causes of roan population decline for the past 30 years in Ruma National Park (RNP), the only park where wild roans remain in Kenya, and (2) predict the probability of roan persistence under existing and alternative management options. PVA was done using long-term data based on population dynamics, life history, climatic conditions, and expert knowledge. Poaching was identified as the main cause of roan decline in RNP. Several antipoaching and prioritized habitat management interventions to promote population recovery and sustainable conservation of roans are described. PVA predictions indicated that, without these interventions, the roan population cannot persist more than 3 decades. Furthermore, ensuring sustainable conservation of roans in RNP will boost tourism in Western Kenyan and thus alleviate poverty in this part of the country. Improved income from tourism will reduce the possible pressures from hunting and give greater incentives for local people to be actively engaged in roan conservation.

## 1. Introduction

One-third of the total population of roan antelope* (Hippotragus equinus)* of about 76,000 is now thought to occur in only 4 countries: Burkina Faso, Cameroon, Zambia, and Tanzania [1]. The East African subspecies* (H. equinus langheldi)* has declined rapidly throughout its range, including Kenya, where it now only survives in Ruma National Park (RNP). Recent roan population decline in RNP from 100 individuals in 1979 to less than 50 currently is of serious concern to the Park management, because a population of less than 50 roan antelopes is not considered viable according to population genetic criteria [2]. Without any interventions, the roan population in RNP will eventually go extinct just as it happened in the other Kenyan protected areas in the past. In fact, the question to ask now is not if but when the extinction will occur. Population viability analysis (PVA) can be used to adequately answer this question and to provide evidence on the likely positive or negative effects of alternative management techniques on roan antelope at RNP.

PVA is a set of modelling techniques that estimates the future size and risk of population extinction [2, 3]. PVA utilizes life history or population growth rate parameters such as survival and fecundity rates as input variables to project dynamics and estimate risk of population extinction [4]. PVA can be used to (i) estimate the probability of extinction [2]; (ii) predict the future population size [5]; and (iii) assess risks and benefits of alternative interventions for population recovery [6].

PVA has been used in the past to model the viability of roan antelope populations and assess consequences of alternative management options [7]. However, some of the findings from these studies are site-specific and cannot be applied generally to areas outside the study area. Hence, further research is needed to devise more effective management interventions for sustainable conservation and management of roan antelopes. This is necessitated by the lack of adequate measures to halt roan population declines in the protected areas previously investigated. The two primary aims of this study were to (i) estimate the future population trends and likelihood of extinction of roan antelopes under the current conditions in RNP and (ii) rank the risks and benefits of management alternatives for roan population recovery.

## 2. Materials and Methods

### 2.1. The VORTEX Baseline PVA Model

There are several computer programs available for PVA, but VORTEX was used in this study as it is widely and commonly used. VORTEX is an individual-based model (IBM) that creates a representation of each animal in its memory and follows the fate of the animal through each year of its lifetime [6]. VORTEX keeps track of the sex, age, and parentage of each animal.

Demographic parameters and life history attributes used for the baseline PVA model were based on findings of previous studies on roan antelopes [7–12]. Complementary information about home ranges and age-specific mortality rates were derived from analysis of roan population and distribution data collected in RNP from 1979 to 2008 [7]. A summary of the specific parameter input data used for the baseline PVA model is presented in Table 1.

### 2.2. Scenarios Modelled

The VORTEX PVA model was used to simulate the population of roan antelopes in Ruma National Park (1) to replicate the observed population decline for past 30 years and (2) to project the future population viability over a 100-year period. In each of these two categories several scenarios were modelled as described below.

#### 2.2.1. Replication of the Observed Population Decline

Simulations of the observed roan population decline for the past 30 years were done to investigate the factors responsible for the decline during that period. The baseline scenario used the roan parameters is described in Section 2.1 above.

All other scenarios were a modification of this baseline scenario. To investigate the effect of inbreeding depression, a model was simulated without the inbreeding depression component. Effects of age-specific mortalities were investigated by simulating several PVA simulation runs using varying mortality rates of 0%, 10%, 20%, 30%, and 40%. Catastrophic effects were studied by considering infrequent severe catastrophes and frequent weak catastrophes. For the infrequent and severe catastrophes scenario, the frequency of each catastrophe in the baseline scenario was halved and the severity was doubled. Likewise, for the frequent and weak catastrophes scenario, the frequency and severity of each catastrophe in the baseline scenario were doubled and halved, respectively. Simulation runs were repeated 1000 times to obtain the most likely population trajectory for each scenario.

#### 2.2.2. Projection of the Future Population Viability

The roan population was simulated to project the population viability for the next 100 years using the baseline scenario and several scenarios of alternative management interventions. Eight management options were investigated: (1) reducing the death of adults; (2) reducing the death of calves; (3) reducing death of subadults; (4) reducing effects of fire; (5) reducing effects of drought; (6) combined interventions consisting of reduction of mortalities and severity of fires and drought; (7) restocking with more roans without changing anything else; and (8) establishing an intensively managed protected sanctuary together with restocking. Again, simulation runs were repeated 1000 times to evaluate the population persistence probabilities for each of these options.  Table 2 gives a summary of the demographic parameters used by different scenarios in comparison with the baseline model. Other parameters were the same as those of the baseline model except age-specific mortalities and impacts of catastrophes on drought, fire, and floods.

Adult mortality could be reduced by increasing security patrols, establishing more security outposts, providing adequate suitable habitat through provision of adequate water supplies, controlling the number of competing grazers in the park, and initiating community development projects to alleviate poverty with the aim of reducing poaching for bush meat as a livelihood (management option 1). Calf mortality could be reduced by maintaining conducive habitat for secluding calves from predation by controlled burning (management options 2). Subadult mortality could be reduced by providing adequate suitable habitat for subadults through provision of enough water supplies and controlling the number of competing grazers in the park (management option 3). Some of the competing grazer species include the reedbuck and hartebeest, whose populations have been shown to correlate negatively with roan population in the park [15–17]. The severity and spread of fires could be reduced by implementing prescribed burning programs and increasing road fire breaks (management option 4). The effect of drought (management option 5) could be mitigated by construction of more water dams and water troughs and ensuring that water is pumped to these water points during the periods of drought. The combined intervention scenario (management option 6) assumed that all the above five interventions were to work simultaneously to reduce age-specific mortalities and effects of catastrophic fires, droughts, and floods. Management option 7 assumed running the baseline model with different initial roan population groups from restocking with 4, 6, 8, and 10 roan groups. The typical herd size for a breeding roan group is 12 individuals [10, 15].

The protected sanctuary (management option 8) would promote roan population recovery via several intensive management strategies: (i) prescribed burning; (ii) construction of more road fire breaks; (iii) construction of more water points and ensuring that water is pumped to these water points during the dry season; (iv) putting more effort in controlling poaching by increasing security patrols and establishing more security outposts; (v) limiting the number of competing grazers in the sanctuary; (vi) removal of predators from the sanctuary; (vii) improving the relationship with the surrounding local communities so that they support wildlife conservation; (viii) initiating community development projects to alleviate poverty with the aim of reducing poaching for bush meat as a livelihood; (ix) providing mineral supplements; (x) offering improved veterinary services; (xi) construction of ridges and trenches to control flooding; (xii) ensuring that the sanctuary is fenced and the fence is maintained in a functioning state; and (xiii) restocking with 4, 6, 8, and 10 roan groups. These strategies could increase the carrying capacity from 288 to 640 roans as has been proved in South Africa [18].

#### 2.2.3. Sensitivity Analysis

The projection of future population viability was repeated to test the sensitivity of age-specific mortalities and catastrophes. A shorter period of 30 years was used as this was considered of relevance to park management objectives at the moment. In the sensitivity analysis, the age-specific mortalities and severity of catastrophic fires and droughts were set from 0% to 40% at intervals of 5%. The population at 30 years for each of these scenarios was analyzed using a generalized linear model (GLM) performed with a Poisson error structure to assess which parameters are most influential in the population viability analysis.

## 3. Results

### 3.1. Replication of the Observed Population Decline

**Figure 1:**
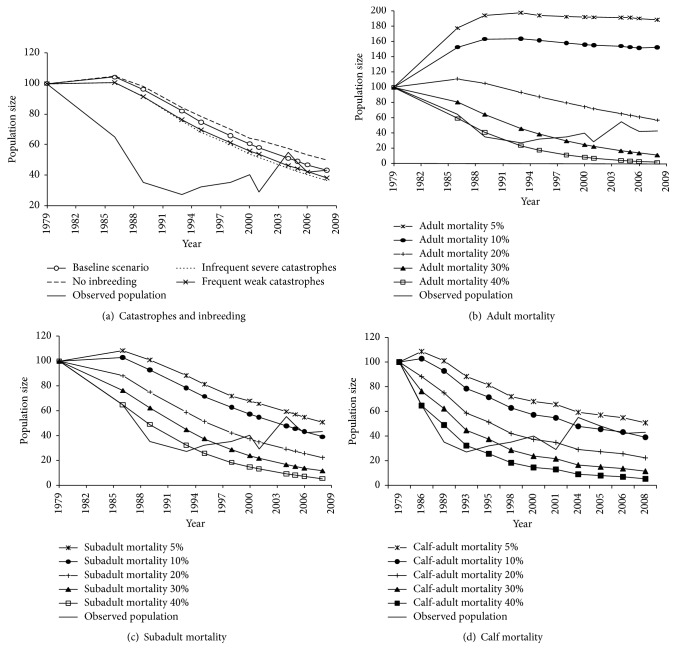
Comparing modelled mean population size with observed roan antelope population in RNP between 1979 and 2008.

The baseline model derived from the observed roan parameters and those obtained from past studies yielded a population of 43, which is the same as the current population of roans remaining in RNP, but the model was different from the observed trend of population decline for the past 30 years (Figure 1(a)). Nevertheless, it can be used as a basis for projecting future population viability.

Models that included inbreeding depression, infrequent severe catastrophes, or frequent weak catastrophes did not differ much from the baseline model (Figure 1(a)). This implies that inbreeding depression and catastrophes were not the major causes of population decline during the past 30 years.

Models with varying age-specific mortality rates seemed to correspond to the observed population in a number of years. The roan population decline from 1979 to 1989 could be attributed to high mortality rates of up to 40% in adults, subadults, or calves. Reduction of adult mortality to 10% and below caused the population to increase accordingly (Figure 1(b)).

However, reduction in mortality rates of subadults and calves to levels as low as 5% did not halt the population decline (Figures 1(c) and 1(d)). This suggests that the observed population decline for the past 30 years may have been primarily controlled by variation in adult mortalities caused by poaching of adult roans and effects of catastrophes such as drought, fire, and floods. Poaching of roans in the study area (RNP) especially using snares has been reported by several researchers over the last 35 years [15, 19–25]. This implies that years with low adult mortality could allow the population to increase and vice versa for years with high adult mortalities. For instance, population increases between 1993 and 2000 and between 2001 and 2004 may reflect a corresponding reduction in adult mortality. In fact the slight increase in roan population observed between 2001 and 2004 was attributed to increased antipoaching security efforts [23, 26].

### 3.2. Projection of Future Population Viability

**Figure 2:**
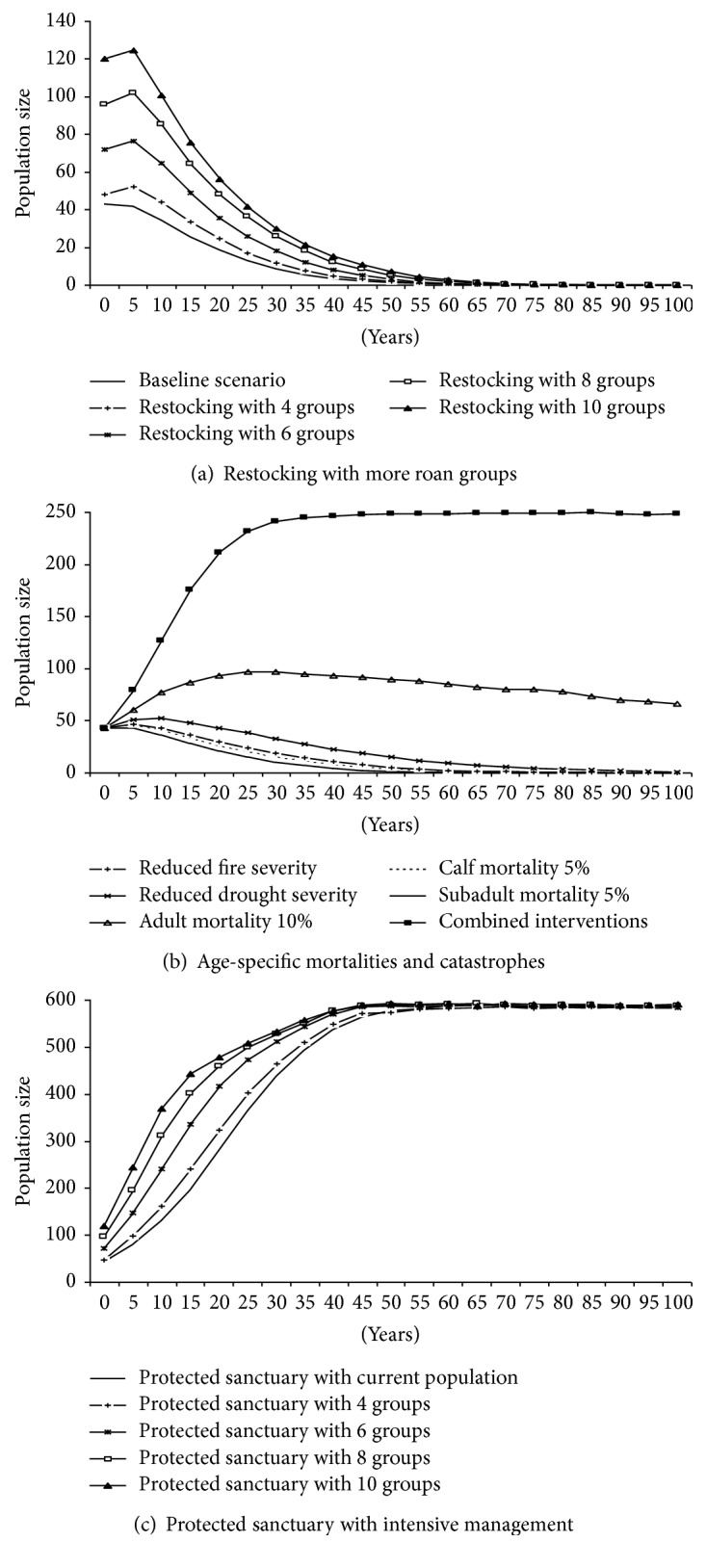
Mean population size for populations simulated over 100 years under alternative management options: (a) restocking; (b) reducing age-specific mortalities and catastrophes; and (c) establishing a roan sanctuary with intensive management.

Under the current situation (as simulated by the baseline scenario), the roan population has a 100% probability of extinction before 100 years. Extinctions are estimated to begin in 32 years (Table 3). Restocking the park with more roan groups can postpone the median time to extinction up to 48 years but it does not lower the probability of extinction (Figure 2(a), Table 3).

Reducing the rates of calf and subadult mortality to 5% did not have any significant change on the probability of extinction or time to extinction as compared to the baseline scenario (Figure 2(b); Table 3). However, reduction of fire and drought severity could reduce the probability of extinction and enable a persistence probability over 100 years of less than 1% and 3%, respectively (Table 3). Reduction of adult mortality to 10% changed the population growth rate from negative to positive; it caused the population to increase at a rate of 0.1% per year. Also, under this management option, the roan population showed a persistence probability of more than 86% over 100 years (Table 3). Combined management interventions could raise the population growth rate to 2.3% and the persistence probability to 99.8% over 100 years.

Intensive management of the current roan population in a protected sanctuary could raise the population growth rate to 4.3% and allow the population to persist for 100 years with a persistence probability of 100% (Table 3). Under this management option, the population will reach the carrying capacity in 65 years.

Restocking with more roan groups combined with the protected sanctuary intervention did not show any improvement in the overall population growth rate apart from reaching the carrying capacity slightly earlier (Figure 2(c)). However, restocking caused the population to reach high numbers within a short period and to reach the carrying capacity faster. For instance, at 10 years, the simulated mean population size was 130 roans for the management option with current initial population of 3 roan groups (36 individuals) and 370 roans for that with an initial population of 10 roan groups (120 individuals). Also, using the current initial population, the simulated mean population size reached carrying capacity in 65 years whereas that with 10 groups reached the same carrying capacity about 20 years earlier.

### 3.3. Sensitivity Analysis

**Figure 3:**
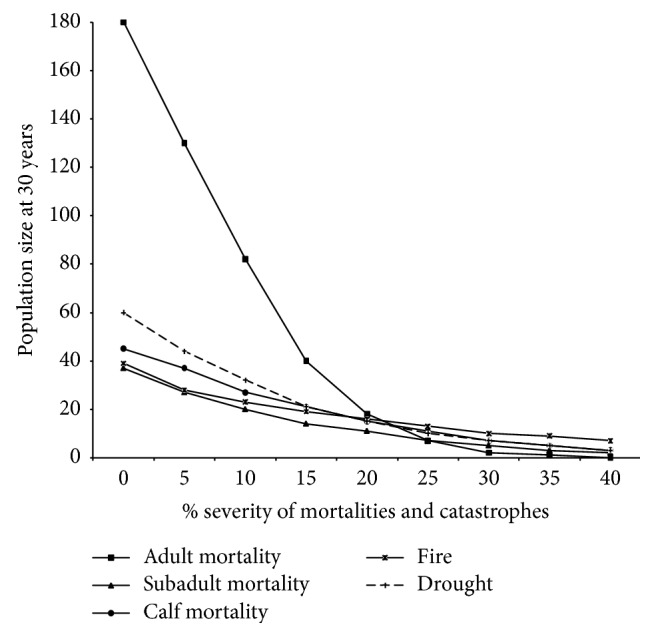
The impact of age-specific mortalities and catastrophes on roan population viability. The mean population size at 30 years was obtained by simulating a PVA model over a 30-year period with an initial population of 43 roans under varying levels of age-specific moralities and catastrophes from 0% to 40%.

Sensitivity analysis was performed for the age-specific mortalities, fire, and drought. Adult mortality was the most sensitive parameter and the most important in determining the change of the roan population (Figure 3). Maintaining adult mortality rates lower than 15% could allow the population to increase and recover. Adult mortality rates higher than 15% could cause the roan population to decrease and consequently go extinct. Maintaining the severity of catastrophic droughts at levels lower than 5% could increase the roan population at a rate lower than that caused by reduction of adult mortalities. The variation in calf and subadult mortalities as well as severity of fires had no substantial impact on the roan population dynamics; whether their effect is eliminated or increased the roan population will eventually decline to extinction.

Further analysis of the impact of these five parameters on roan population dynamics using generalized linear models showed that the adult mortality could account for 78% of the population decline (Table 4). All the other four parameters together accounted for only 18% of the decline. Examination of the regression coefficients (*β*) showed that drought was the second important factor responsible for roan population changes.

## 4. Discussion

### 4.1. Replication of the Observed Population Decline

Simulation to replicate the observed roan population decline helped to understand the factors responsible for the decline. Our results indicate that the most important factor affecting the roan antelope population is adult mortality, which confirms existing concerns about the increasing amount of poaching within the park. Records show that poaching in the park was heavy as early as 1970s [19] and has continued until now [22–25]. The highest rate of population decline from 1979 to 1989 coincided with a period of transition from National Reserve to National Park status [27]. This transition may have caused escalation of poaching by the local community in retaliation to eviction from the park. In Kenya, a national reserve allows limited access of its resources to local communities but national parks are strictly managed for wildlife with total exclusion of humans. Park records also show that the slight increase in roan population observed between 2001 and 2004 was attributed to increased antipoaching security efforts [23, 26]. Past studies have shown that high levels of predation, especially of adults, were responsible for roan population decline in Kruger National Park [7, 9, 25].

### 4.2. Projection of Future Population Viability

Projections of future roan population viability based on the baseline scenario showed a high probability that at least three decades could pass before extinction eventually occurred, even if no intervention is undertaken. However, this should not send the wrong signal that interventions are not urgently needed since extinctions have been projected to happen in the near future. In Masai Mara National Reserve, a remnant roan population of 45 (±17 SE) animals in 1971 became extinct after about two and half decades [28]. Therefore, the predicted extinction of the RNP remnant roan population of 43 animals in about three decades seems realistic. Hence, this period should be used to implement management interventions.

The projections of future population viability indicated that various management interventions can halt the population decline and cause the population to recovery to healthy levels and probably to carrying capacity. These include reduction of adult mortality, combined management interventions, and establishment of an intensively managed protected roan sanctuary. Reduction of adult mortality appears to be the most crucial intervention without which the roan population is destined to go extinct in the future. Measures for reducing adult mortality were included as part of the combined interventions and protected sanctuary management options. Adult mortality needs to be maintained at levels far below 15% for the roan population to recover. Studies in South Africa have found out that adult mortality rates of 15% or more severely restricts the recovery potential of roan antelopes [7].

Unfortunately, some management interventions such as prescribed burning, construction of more water points, and restocking under the current conditions could be a waste of time, effort, and money. Although these interventions are still important and necessary they can only be effective when combined with measures of reducing adult mortality.

### 4.3. Management and Conservation Implications

The next crucial stage after identification of the cause of roan population decline in RNP is to implement effective strategies that can halt the decline and propel the population to recovery. The high adult mortality rate on roan antelopes in Kenya is most likely caused by poaching [15, 19–24] and not by predation. There are no lions in RNP and the only potential predators are hyenas, which may prey on calves and not adult roans [29]. Therefore, the best interventions in this case should include methods of reducing adult mortality by controlling poaching in RNP. Such methods can involve increasing security patrols, opening more security outposts, involving the surrounding communities in management of the park, and initiating community development projects to alleviate poverty with the aim of reducing poaching for bush meat as a livelihood. Involvement of local communities has proven to be effective in curbing elephant poaching in Tanzania's Selous ecosystem [30], reducing rhino poaching in Kenya's Lewa Conservancy [31], enhancing conservation of many wildlife species in Kenya's Nakuru Wildlife Conservancy [32], and reducing human-wildlife conflicts in Kenya's nonprotected areas [33].

Apart from adult mortality, the decline in roan population was also contributed by other factors such as drought, fire, and calf and subadult mortality [16, 34, 35]. Therefore, for sustainable conservation of roans in RNP to be achieved, a combined management intervention seems to be the way forward. Such intervention could include (i) measures of reducing adult mortality, (ii) establishment of more water points to alleviate effects of drought, (iii) prescribed burning to control the effects of fires, and (iv) more active manipulative management of the roan habitat. For instance, while reducing adult mortality to 10% could increase the current roan population in RNP to about 100 individuals in 30 years, employing a combined management option could propel the population to about 250 individuals over the same period.

With the current roan habitat conditions coupled with the observed habitat decline over the past 30 years, interventions in RNP under the prevailing management regime may not manage to propel the roan population to numbers that can be considered viable in isolation. Furthermore, putting all efforts of population recovery of a critically locally endangered species in one isolated population is too risky. There is need to establish other roan populations in Kenyan protected areas, where they became locally extinct in the past. Establishing an intensively managed protected sanctuary for roan antelopes in RNP can provide a “seed population” that can be used to establish several other populations in the roan's former known ranges in Kenya. This may offer a more lasting solution to the problem of roan population decline in Kenya. This concept of breeding roan antelopes in an enclosed sanctuary has already proved to be very successful in other countries [7]. In South Africa, 7 roans were placed in a fenced enclosure within the roan range in 1994 and the population grew to 41 roans by 2001 [7].

## Figures and Tables

**Table 1:** Biological and ecological attributes of roan antelopes used as input data in the baseline scenario to the VORTEX model. The percentage impact of catastrophes (drought, fire, and floods) indicates how much the reproduction, survival, or frequency is reduced from what typically it should be.

Demographic parameter	Value	Source/reference
Breeding age females	3 years	[7]
Breeding age males	5 years	[11]
Mating system	Polygamous	[11]
Percent of adult females in breeding pool	100%	[8]
Percent of adult males in breeding pool	30%	[8]
Maximum litter size	2 calves	[13]
Mean litter size	1 calf	[11]
Inbreeding depression		[4, 14]
Lethal equivalents	3.14	
Percent due to recessive lethal alleles	50%	
Reproduction active life	12 years	[11]
Age classes		[9]
Calves	0-1 years	
Subadults	1-2 years	
Adults	>2 years	
Annual survival rates:		[8]
Calves (mean ± SD)	89 ± 2%	
Subadult (mean ± SD)	92 ± 3%	
Adult (mean ± SD)	79 ± 5%	
Birth rate per female per year (mean ± SD)	0.45 ± 0.15	[8]
Sex ratio at birth	1 : 1	[11]
Carrying capacity (mean ± SD)	288 ± 20	[8]
Multiplicative impact of catastrophes		[8]
(i) Drought		
(a) Frequency	20%	
(b) Reproduction	10%	
(c) Survival	15%	
(ii) Fire		
(a) Frequency	20%	
(b) Reproduction	5%	
(c) Survival	5%	
(iii) Floods		
(a) Frequency	10%	
(b) Reproduction	0%	
(c) Survival	15%	

**Table 2:** *Demographic parameters of roans used by different PVA scenarios in comparison* with the baseline model. All other parameters are the same as those listed in Table 1. The scenarios/management options are (1) reducing adult mortality; (2) reducing calf mortality; (3) reducing subadult mortality; (4) reducing effects of fire; (5) reducing effects of drought; (6) combined interventions; (7) restocking with current scenario; (8) protected sanctuary together with restocking. The values in bold are those different from the baseline model.

Demographic Parameter	Baseline model	Scenario 1	Scenario 2	Scenario 3	Scenario 4	Scenario 5	Scenario 6	Scenario 7	Scenario 8
Mortality									
Calves	11%	11%	**5%**	11%	11%	11%	**5%**	11%	**5%**
Subadult	8%	8%	8%	**5%**	8%	8%	**5%**	8%	**5%**
Adult	21%	**10%**	21%	21%	21%	21%	**10%**	21%	**10%**
Number of breeding								**4, 6,**	**3, 4, 6,**
groups @ 12 roans	3	3	3	3	3	3	3	**8 & 10**	**8 & 10**
Carrying capacity (mean ± SE)	288 ± 20	288 ± 20	288 ± 20	288 ± 20	288 ± 20	288 ± 20	288 ± 20	288 ± 20	**640**
Multiplicative impacts of catastrophes									
(i) Drought									
(a) Frequency	20%	20%	20%	20%	20%	20%	20%	20%	20%
(b) Reproduction	10%	10%	10%	10%	10%	**5%**	**5%**	10%	**5%**
(c) Survival	15%	15%	15%	15%	15%	**5%**	**5%**	15%	**5%**
(ii) Fire									
(a) Frequency	20%	20%	20%	20%	**10%**	20%	**10%**	20%	**10%**
(b) Reproduction	5%	5%	5%	5%	**2%**	5%	**2%**	5%	**2%**
(c) Survival	5%	5%	5%	5%	**2%**	5%	**2%**	5%	**2%**
(iii) Floods									
(a) Frequency	10%	10%	10%	10%	10%	10%	**5%**	10%	**5%**
(b) Reproduction	0%	0%	0%	0%	0%	0%	0%	0%	0%
(c) Survival	15%	15%	15%	15%	15%	15%	**10%**	15%	**10%**

**Table 3:** Results of the VORTEX PVA model for roan antelopes simulated over 100 years under alternative management options.

Management option	*r*	SD (*r*)	PE (%)	*N*	SD (*N*)	TE (years)
Baseline (no action)	−0.074	0.186	100	0	0	32
Restocking with 4 groups (option 7a)	−0.07	0.185	100	0	0	36
Restocking with 6 groups (option 7b)	−0.07	0.178	100	0.01	0	42
Restocking with 8 groups (option 7c)	−0.071	0.173	99.9	0.01	0.19	45
Restocking with 10 groups (option 7d)	−0.07	0.171	99.8	0.02	0.22	48
Reduced fire severity (option 4)	−0.055	0.178	99.2	0.12	1.41	43
Reduced drought severity (option 5)	−0.044	0.164	97.2	1.05	2.77	53
Calf mortality 5% (option 2)	−0.061	0.183	99.9	0	0.06	38
Subadult mortality 5% (option 3)	−0.068	0.185	99.9	0	0.06	35
Adult mortality 10% (option 1)	0.001	0.134	13.7	66.37	49.27	>100
Combined interventions (option 6)	0.023	0.099	0.2	248.3	31.58	>100
Protected sanctuary with current population of 3 groups (option 8a)	0.043	0.095	0	582.13	62.76	>100
Protected sanctuary with 4 groups (option 8b)	0.043	0.096	0	586.38	64.12	>100
Protected sanctuary with 6 groups (option 8c)	0.042	0.095	0	586.27	65.38	>100
Protected sanctuary with 8 groups (option 8d)	0.041	0.095	0	588.45	56.91	>100
Protected sanctuary with 10 groups (option 8e)	0.041	0.094	0	590.99	61.08	>100

NB: *r* and SD (*r*) = population growth rate and its standard deviation; PE (%) = mean probability of extinction; *N* and SD (*N*) = mean population size and its standard deviation; TE = median time to extinction, in years.

**Table 4:** Relative importance of age-specific mortalities and catastrophes on roan population viability. The parameters *R*^2^ (coefficient of determination) and *β* (regression coefficient) and AIC are derived from generalized linear models (GLM).

Parameter	*R* ^2^	*β*	AIC
Adult mortality	78.3%	−4.096	71.89
Subadult mortality	3.7%	−0.810	138.84
Calf mortality	3.4%	−0.925	138.96
Fire	4.8%	−0.738	138.33
Drought	6.1%	−1.257	137.68
